# A skill to be worked at: using social learning theory to explore the process of learning from role models in clinical settings

**DOI:** 10.1186/s12909-018-1251-x

**Published:** 2018-07-03

**Authors:** Jo Horsburgh, Kate Ippolito

**Affiliations:** 1 Educational Development Unit, Imperial College London, Level 5, Sherfield Building, Exhibition Road South Kensington, London, SW7 2AZ UK; 2 Department of Primary Care and Public Health, Imperial College, London, UK

**Keywords:** Role modelling, Social learning theory, Clinical teaching, Bandura, Observation, Reinforcement

## Abstract

**Background:**

Role modelling is widely accepted as being a highly influential teaching and learning method in medical education but little attention is given to understanding *how* students learn from role models. This study focuses on role modelling as an active, dynamic process, involving observational learning and aims to explore the process involved, including strategies that learners and medical teachers use to support this.

**Methods:**

To gain insight into medical students’ and clinical teachers’ understanding of learning through role modelling, a qualitative, interpretative methodology was adopted, using one-to-one semi-structured interviews. Six final year medical students and five clinical teachers were purposefully sampled and interviewed. Interviews were audio recorded and transcribed. The data were then analysed using open and axial coding before codes were combined to develop broader themes.

**Results:**

Students could identify ways in which they learnt from role models but acknowledged that this was complex and haphazard. They described selectively and consciously paying attention, using retention strategies, reproducing observed behaviour and being motivated to imitate. Students evidenced the powerful impact of direct and vicarious reinforcement. Clinical teachers reported using strategies to help students learn, but these were not always consciously or consistently applied or informed by teachers’ understanding of their students’ cognitive processing.

**Conclusion:**

Findings illustrate in what ways the process of learning from role models in clinical settings is challenging. They also support the relevancy and usefulness of Bandura’s four stage social learning model for understanding this process and informing recommendations to make learning from role modelling more systematic and effective.

**Electronic supplementary material:**

The online version of this article (10.1186/s12909-018-1251-x) contains supplementary material, which is available to authorized users.

## Background

Learning from role models is widely accepted as being an influential medical educational method, especially during clinical rotations [1, 2]. Park et al. argue that of all learning experiences exposure to clinical environments and the role models present there have ‘the greatest impact on professional formation’ ([2], p.134). Despite this perceived value, we would suggest that the term ‘role model’s’ common but vaguely defined usage [3], coupled with limited awareness about the process of learning from role models, lessens the value of this construct as a way of explaining how medical students learn in clinical settings. The concept of ‘role model’ draws on two theoretical constructs. First, the tendency of people to identify individuals who hold a social position to which they themselves aspire, in this context a successful senior medical student or well-regarded consultant in a specialty of interest. Second, the concept of modelling, or social learning [4], which suggests that individuals pay attention to role models because they believe they can learn skills and accepted ways of behaving in a particular context [5]. Although we see the relevancy of both, our study is framed by the latter because we perceive a need to critically examine the active, dynamic process between role model and observer, or teacher and learner.

Most previous studies in this area have focused on *what* students learn from role models and there is much consensus on the attributes of positive doctor role models including excellent clinical knowledge and skills, patient-centred approach and humanistic behaviours such as empathy and compassion [6, 7]. Our interest lies in further examination of *how* students learn from role models in order to maximise the conditions for this type of learning. This builds on the work of Cruess et al. [8] who proposed the idea of making explicit the implicit reasons behind role models’ actions for the benefit of both learners and role models. In addition, the BEME Guide no. 27 [9] highlighted the importance of recognising the process of learning from role models. In this paper we take an exploratory approach to gain more insight into medical students’ and clinical teachers’ understanding of how individuals learn through behavioural observation and role modelling. Based on this insight we will attempt to suggest ways to improve learners’ and teachers’ understanding and therefore the effectiveness of this method.

### Bandura’s theory of social learning

Bandura’s theory of social learning [1] provides a useful framework for us to consider how students learn via observational learning and modelling. For Bandura, learning takes place in a social setting via observation, but it also involves cognitive processes; that is, learners internalise and make sense of what they see in order to reproduce the behaviour themselves. Gibson argues that this involves ‘the psychological matching of cognitive skills and patterns of behaviour between a person and an observing individual’ [3, 5]. Bandura proposed that this type of learning involved four different stages – attention, retention, reproduction and motivation.

The first stage is *attention* whereby learners need to attend to the behaviour. They need to actually see the behaviour that they want to reproduce or that others want them to reproduce. Secondly they need to internalise and *retain* what they have seen. This involves cognitive processes in which a learner mentally rehearses the behaviour or actions that are to be reproduced. Thirdly they need opportunity to *reproduce* the behaviour by converting the information obtained from attention and retention processes into action. Finally learners need to be *motivated* to enact or imitate the behaviour they have observed. This motivation occurs via reinforcement, of which Bandura proposes three different types – direct reinforcement, vicarious reinforcement and self-reinforcement.

We wanted to better understand to what extent learners and teachers are aware of and consciously make use of the underlying cognitive processes described by Bandura, even though they are unlikely to be aware of his model, to maximise learning from role modelling and what they think creates barriers to this four-stage process. In particular we wanted to investigate:

- What are the processes occurring in clinical settings that support learning from role models and what hinders it?
- What approaches do learners take to analyse, evaluate and adopt or reject what they learn through observation of and engagement with role models?
- What strategies do role models consciously apply to encourage their learners to learn in this way?

## Methods

A qualitative, interpretative methodology was adopted, with one to one semi structured interviews being conducted. Six final year medical students (Anita, Mark, Pete, Emily, Jason and Liam), and five clinical teachers (Shivani, Melanie, Iris, Stefan and Abigail), identified here by pseudonyms, were purposefully sampled on the basis that they would provide rich insight into teaching and learning in clinical settings [10] The clinical teachers were from a variety of specialities and had a range of teaching experience. The aim was not to achieve theoretical saturation but to gain in-depth insight into 11 individuals’ unique experiences to better understand how learners and teachers perceive and make sense of learning from role modelling in clinical settings [11]. The two interview guides varied slightly between students and teachers to take into account their differing roles. Although we were aware of Bandura’s social learning theory and anticipated it would help us to interpret our data his four predetermined categories did not influence the question design, which were deliberately broad, open questions that allowed interviewees to describe the process of learning in a clinical context in their own words (see Additional file 1). Interview questions were not validated but were piloted for understanding. Interviews were audio recorded and transcribed. The data were analysed using open and axial coding [12] which was completed by both authors independently before codes were combined to develop broader themes. For the purposes of this paper we have adopted a theoretical data analysis approach; identification of themes was guided by our specific research questions and our theoretical interest in the relevancy of Bandura’s four stage model for analysing learning in the clinical setting. This led to more detailed analysis of particular aspects of the data rather than rich description of all data collected [13] (see Table 1 below). Ethical approval for this study was gained from the Medical Education Ethics Committee at Imperial College, London.

**Table 1:** Codes, themes and example quotes from student and clinical teacher interviews

Codes	Themes	Example quotes	Bandura categories
Permission to participate	Being present and involved	‘And he [the consultant] was like fine, go and integrate yourself into the team’ (Pete, student) ‘I think a lot of the time as a medical student you do stand there feeling like a lemon’ (Anita, student)	Attention
Permission to ask a question/for advice			
Being given a role			
Feeling included			
Wasted time			
Preparing themselves			
Contact with role models	Continuity of and exposure to role models	‘You find people that you work with well and then you have to start again every three or four weeks’ (Anita, student) ‘I also try and highlight anything I thought might have been particularly helpful about the consultation, a particular sign or something or a particular… you know something that the patient asked me that was difficult to answer.’ (Melanie, teacher)	
Role models being approachable			
Role models being observable			
Valued knowledge	Aligned values	‘When you find a good role model it tends to be someone you can relate to.’ (Liam, student) ‘when I see patients react that way [positively] that gets me more that ‘oh, I’ve published 70 things’ (Emily, student)	
Getting along			
Attitude to patients			
Attitude to colleagues			
Value to patients			
Value to colleagues			
Making meaning	Learning the language	‘Because you hear people present….and in the first year you’re like’ I don’t know what they are talking about’ but the more you listen to it and the more you look things up… you just absorb it’ (Anita, student) ‘You need to use the right words…a good role model will teach you how to make yourself just sound better’ (Liam, student)	Retention
Looking up terminology			
Discussing meaning with peers			
Using the right words			
Sounding professional			
Role models thinking/reasoning out loud	Understanding thought processes	‘So if they can talk through their thought process then it’s easy for you to kind of understand it better that way’ (Liam, student) ‘They might say, for example, ‘I saw a patient with such and such a presentation… ‘what would your thought process have been?’ (Melanie, teacher)	
Modelling clinical thinking			
Similar thought processes			
Formal reflection requirement	Meaningful reflection	‘Hearing a good question and going ‘that’s a really effective what of asking that. Mine’s not as good. I’ll try and see if it works for me’ (Jason, student) ‘If you enforce people to reflect on a price of paper…. They’re not going to do it.’ (Stefan, teacher)	
Student led reflection			
Value of reflection			
Impact of reflection			
Curricula alignment			
Student-led note taking	Writing it down	‘I’ll write done the date and ‘ok, this is what I’ve taken out of it’ (Liam, student) ‘get them to write down a few things that they may have noticed me doing a lot or questions that they noticed me… asking a lot and then try and get them to reflect on why’ (Melanie, teacher)	
Teacher-directed note taking			
Sharing notes			
Priority of exams	Barriers to retention	“They’re saying you can get a lot more out if you go on Pastest (online revision resource), there’s 3000 questions and you spend a day doing that, that’s much more useful then writing notes on a ward round.” (Mark, student).	
Practice with peers	Opportunity to practice	‘I think you have to be proactive…either asking for teaching or….if there are any spare patients that it would be good for me to examine’ (Anita, student) ‘We don’t necessarily want the knowledge now, we want to practise being baby doctors’ (Pete, student)	Reproduction
Competition for practice opportunity			
Proactivity			
Practice with patients			
Practice being in setting			
Role model’s feedback	Feedback	‘So she [role model] got me to be the person doing the questions, taking the history etc.… and then she’d either top up or said’ well done… [because] that…[gives] a very good indicator of where you are at…’ (Jason, student)	
Peers’ feedback			
Lack of continuity of feedback giver			
Wanting to please	Feedback	‘So I…didn’t want to disappoint him [role model] and I did actually care what he thought’ (Mark, student) ‘When the patients come in I’ll leave you alone to do that because I know having spent the past half day that you are competent in this…’ (Liam, student)	Motivation
Being considered more independent			
Response or reaction of students	Observing others’ responses - Vicarious reinforcement Vicarious punishment	‘I think perhaps the negative behaviours have a greater impact… when they [students] have observed something they don’t want to emulate or that sits uncomfortably with them, they do…tell each other’ (Iris, teacher) I’d always thought that if you want kids’ attention you want to be loud but to him [role model] is was the opposite. If he kept quiet they’d do the same to him’ (Liam, student)	
Reaction of colleagues			
Reaction of patients			
Lack of opportunity to see reaction			
Clinical outcomes			
Being considered more independent			
Value to them	Reciprocity	‘If you have a good role model it encourages you to be a role model for other people if you can…it’s like paying it forward’ (Liam, student) ‘They’re [students] are in a good position to see what I’m doing and if I would have done anything better…’ (Shivani, teacher)	
Two way			
Feedback to role models			

## Results

For the sake of clarity and to better explore the process of learning, in this paper we have chosen to emphasise the learner experience and perspective, with data from the clinical teachers used to enhance and further illuminate the students’ viewpoints. Table 1 shows the codes and themes derived from analysis of the interview transcripts, illustrative quotes for each of the themes, and the process from Bandura’s theory that these relate to.

The students interviewed were able to identify ways in which they learnt from role models but acknowledged that this was a complex and haphazard process.

## Discussion

Having presented the codes, themes and illustrative quotes from the interview transcripts, these will now be discussed in further detail.

### Attention

#### Being present and involved

Despite needing to be physically present and able to see the action some students reported feeling in the way like ‘lemons’ or ‘ghosts’, suggesting they needed their participation to be legitimised. They described that legitimacy as coming from being given a specific role, such as taking a history from a patient. Anita, for example, described being asked by a consultant to sit and chat with a patient whilst she ate her breakfast. For the student this proved to be a rich and memorable learning experience. This purposeful role gave her an involved perspective from which to observe and maintained her attention. Lave and Wenger [14] refer to the way that newcomers to a community of practice learn by participating as *legitimate peripheral participation*. But opportunities for *legitimate peripheral participation* need to be created for medical students and the value of this should not be underestimated as the quotes from students’ demonstrated.

Teachers were also aware of this need to actively involve students, particularly those less confident ones, but due to time pressures this was not always possible. Most teachers agreed that some role models were easy to identify - what Abigail referred to as ‘superstars’. But there was an acknowledgement that other role models might be more useful, particularly in the early stages. Therefore, as has been found in other research [8] signposting less obvious behaviours, such as at what point and how junior doctors involve senior colleagues was important.

#### Continuity of and exposure to role models

Barriers to paying attention included lack of continuous exposure to any one role model or patient, meaning that students could not truly analyse role model’s behaviours or evaluate the impact of that behaviour on others. Attending to patterns in role models’ practice takes time and for some the short phases that tend to characterise medical students’ clinical rotations made this difficult.

Where role models had facilitated more continuous observations of their practice the educational value of this could be recognised by students. For some students, their experience aligned well with Gioia and Manz assertion that “if an observer is to learn effectively from a model it is important for the model to be credible, reasonably successful, clearly display the behaviour to be learned, and otherwise facilitate the attention process.” ([15]: 528).

Teachers also commented on the fragmented nature of clinical rotations. Difficulty in identifying patterns in behaviour and forming relationships created by lack of continuous exposure to role models seemed to be disruptive and demotivating for both students and teachers. Being aware of these challenges to attendance and opportunities to observe was important. Faculty also needed to be present in order for students to observe their practice, although as Iris pointed out, clinical teachers were still role modelling even if absent.

#### Aligned values

The students reported paying close attention when they observed a behaviour that aligned with their views of what was important about being a doctor. For Emily, the positive reactions she observed from her role model’s patients were more important than them having a long list of publications.

The artificial separation of scientific and medical knowledge from skills and attitudes within medical curricula can be confusing and students saw clinical rotations as a place to learn how to bring these elements of a doctor’s practice together, although they found it difficult.

### Retention

There is an enormous amount for a learner to take on board when in a clinical setting and they cannot possibly be expected to retain everything they observe. In order to avoid becoming overwhelmed learners seek cues to work out what is important to retain and develop strategies for doing so.

#### Learning the language

Students spoke about comprehending and retaining the unfamiliar clinical language that they heard their role models use. This sometimes involved looking it up later or consulting peers.

Particularly useful role models deliberately helped students to learn the language and develop the way they communicated in the clinical setting.

#### Understanding thought processes

Students talked about how they valued their role models giving insight into their thought processes as this enabled them to understand the reasoning behind the behaviours they were observing, including coping with uncertainty, and helped them to make sense of and retain the particular learning point.

Liam, who like other students talked about the importance of being able to relate to their role models, attributed this relatability, in part, to him and his role models thinking alike. This seems connected to the point made earlier about the attractiveness of aligned values between role models and observers.

#### Meaningful reflection

Reflection is widely acknowledged as aiding development, but how do learners make use of reflection when learning from their role models? Even though Jason claimed not to be ‘a fan of formal reflection’ he had clearly developed a critically reflective approach to help him extract personal value from what he had observed and imitate aspects of it before deciding what to retain.

Stefan also talked about the importance of authentic reflection and the role of teachers in creating space and support for students to evaluate what has been observed in clinical settings.

#### Writing it down

Liam described a particularly systematic approach to aid retention and processing of what had been observed, clearly guided by his role model.

Such strategies were encouraged and signposted by teachers, with Melanie referring to use of an advanced organiser [16] to help students consciously retain what they observed. She described an example in which she asked students who were observing her on a busy labour ward to write down a few things they noticed her doing or questions she asked the patient and then, importantly, got them to reflect on why they noticed these specific things or why they thought them important. Facilitating this metacognitive process, whereby students are required to think about what and how they are learning through observation, may also enable teachers to reinforce or ‘correct’ important take away messages.

### Reproduction

#### Opportunity to practice

The opportunity for hands-on practice has been reported as lacking from some clinical-based learning experiences [17] In our study students talked about being given the opportunity to put into practice the behaviours and strategies that they had observed in their role models. Some needed help to recognise opportunities or to be given permission to take advantage of opportunities and to participate in a legitimate and meaningful way.

Giving students opportunity to legitimately participate in the team may involve considering the roles and expectations of the existing clinical team.

Most students recognised the need to be proactive about identifying and creating their own opportunities for practice and some had strategies for arranging these.

Students’ also highlighted the value of being supported by a role model to identify in advance, in a systematic way, tasks and skills that they could learn through modelling and observation with opportunity for practice.

#### Feedback

When referring to opportunities to put into practice the techniques that they had observed, students highlighted the value of feedback that both reinforced desired behaviours and suggested aspects for development, especially where it was highly contextualised and immediate.

Shivani talked about making use of the student perspective and adopting a more dialogic approach to feedback [18] on what has been observed in way that could offer suggestions for development for both teachers and students.

### Motivation

#### Feedback

Finally Bandura argued that if students are to learn from and reproduce the behaviours that they observe in their role models they need to be motivated to do so. For many students this was a question of direct reinforcement, whether this was a self-regulated process involving perceptions of ‘wanting to please’ or further reinforced by direct, positive feedback, including more independence.

#### Observing other’s responses - vicarious reinforcement and punishment

Two further interesting and useful concepts from Bandura’s social learning theory are vicarious reinforcement and vicarious punishment. Bandura proposed that when observing others we not only learn from their behaviour but also from the reactions of other people to the role model’s behaviour. This is potentially a very efficient way to learn as it allows us to learn from others’ mistakes. Our student interviewees identified a number of examples of being vicariously reinforced or punished and described how the reactions of patients, colleagues or fellow students influenced their decisions to reproduce behaviours they observed. For example, Liam, who was vicariously reinforced having closely observed this paediatrician, chose to adopt his communication technique as a result of the calming effect it appeared to have on children.

Conversely an example of vicarious punishment refers to what Jason considered to be brusque treatment of a patient. He was vicariously punished by the interaction between a role model and patient and as a result talked about wanting to deliberately avoid reproducing this behaviour in his own practice because of the patient’s reaction.

Jason also highlighted barriers that interruptions in exposure to patients and clinicians poses for students wanting to convert observation into practice. It appears to be important to create opportunities for students to observe outcomes of interactions (or for them to be discussed), as well as seeing the behaviour that led to them. Students reported receiving mixed messages about appropriate behaviour through vicarious reinforcement. As Iris commented the less desirable behaviours observed in clinical settings can have a powerful influence, a view supported by Gibson [5], who highlights the value of learning from negative traits as well as positive aspects of role models. Furthermore Bucher and Stelling [19] found, to their surprise, that rather than identifying complete roles models amongst their senior colleagues, as had been assumed, medical students actively identified specific attributes to emulate and to reject, in a process of creating a vision of their ‘ideal selves’. Clinical teachers recognised that students made decisions about who were useful role models on the basis of vicarious reinforcement. in the form of successful clinical outcomes, and/or positive reactions from patients and colleagues. Stefan spoke about how students might use clinical outcomes to judge the value of a particular behaviour when deciding whether to adopt or adapt them.

#### Reciprocating

Student also saw satisfaction and reward in being part of the reciprocal role model cycle themselves and referred regularly to the culture of peer support in medical school.

In terms of closing the reciprocal loop Liam, for example, also talked about how he sent a letter, email or card to his role models to thank them.

However, in general it is unclear how aware role models are of the influence they have on those observing them and indeed how they could be more effective. Clinical teachers commented that they seldom received direct feedback on the impact of their role modelling but Shivani recalled that students had commented on how she had interacted with a patient thus highlighting for her the value medical students derived from being able to closely observe a role model in action. Feedback on how role models have influenced those around them is potentially an important of untapped source of evaluation data.

### Limitations

Whilst this paper has emphasised the benefits of modelling and observational learning, students also highlighted the limitations. This included that the ability to imitate the actions of others and carry out clinical tasks might not be accompanied by underpinning clinical knowledge or rationale in the mind of the learner.

Another limitation is created by the lack of constructive alignment [20] between the formal undergraduate medical curricula, often with an emphasis on gaining knowledge and exam-based assessment, and the authentic, skills-based learning in the clinical setting. This resulted in some learners prioritising revision for their exams over taking the opportunity to learn from observation in the clinical setting.

Finally the unfamiliar and haphazard nature of observational learning opportunities in the clinical settings proved challenging for students to identify and follow to their logical conclusion thus limiting the learning process that Bandura describes. Even when student interviewees described successful learning having taken place it became apparent that they were often not in control of, or even conscious of, the process occurring, let alone able to guide themselves through the four stages identified by Bandura.

## Conclusion

We acknowledge that the exploratory inquiry presented here is a work in progress that does not does not yet reflect the application of a finished construct to an empirical study. However, this small sample of medical students’ and clinical teachers’ insightful accounts of how observational learning from role models happens, leads us to make the following tentative conclusions.

The way students and clinical teachers described learning in this context can be aligned with the four stage model set out by Bandura. That is, participants clearly illustrated how they benefitted from (and felt motivated by) being able to observe or attend to the behaviours of their clinical teachers, being helped to retain new understanding, opportunities to practise the actions or behaviours observed, as well as how they learnt through vicarious reinforcement from seeing the reactions of others. Barriers to learning identified can also be analysed in terms of where these four stages could not be carried out or were interrupted.

Although when asked student and teacher participants demonstrated good understanding of ways in which individuals learn from role modelling,, all participants illustrated that learning from role models in clinical settings is complex and challenging and the processes they described as supporting that learning were often fragmented and inconsistent. Furthermore there appeared to be limited awareness of underlying cognitive processes supporting observational learning. Whilst some of the clinical teachers interviewed did identify methods that they used to enhance observational learning in clinical settings, it could be argued from these findings that this could be done in a more conscious and consistent way.. Whilst consistency is not the ultimate goal in undergraduate learning during clinical rotations and variety in approaches to role modelling adds richness and authenticity to the experience, we have given insight into how a lack of structure can be problematic for students. We believe that, the process of learning from role models in clinical settings is a skill to be worked at. Students in the study demonstrated different levels of awareness and capacity in this regard, and some suggested it would have been useful to be aware at the beginning of their clinical rotations.

On the basis of our theoretically-informed analysis we would like to suggest the explicit use of Bandura’s model to develop a two pronged approach to supporting students’ learning from role modelling. Firstly, by introducing students to Bandura’s four stage model and strategies outlined above to inform development of their skills for maximising their own learning at each stage and their agency within the unfamiliar learning environment. Secondly, by using it to develop teachers’ understanding of how learning from observation occurs and their ability to maximise opportunities and create the conditions in the environment that enable learners to:

- Closely and repeatedly observe role models’ actions and behaviours and patients and colleagues’ responses to these behaviours.
- Be given insight into the invisible thought processes behind the behaviours they observe
- Be given permission and structured opportunity to reproduce and test out observed behaviours in practice and reflect on this.

By linking our exploration of learners’ and teachers’ understanding of how observational learning happens in clinical settings with Bandura’s four stage model we hope to have provided a feasible and memorable framework to guide teachers and students in making more effective use of modelling and observational learning.

## Additional file

Additional file 1: Interview schedule – interview questions for both student and clinical teacher participants. (DOCX 13 kb)

## Abbreviations

BEME: Best Evidence Medical Education

## References

[1] Irby DM (1986). Clinical teaching and the clinical teacher. Acad Med.

[2] Park J, Woodrow SI, Reznick RK, Beales J, MacRae HM (2010). Observation, reflection and reinforcement. Surgery faculty members; and residents’ perceptions of how they learned professionalism. Acad Med.

[3] Jung J (1986). How useful is the concept of role model? A critical analysis. J Soc Behav Pers.

[4] Bandura A (1977). Social learning theory.

[5] Gibson DE (2004). Role models in career development: new directions for theory and research. J Voc Behav.

[6] Wright S, Carrese J (2002). Excellence in role modelling: insight and perspectives from the pros. Can Med Assoc J.

[7] Althouse L, Stritter F, Steiner BD (1999). Attitudes and approaches of influential role models in clinical education advances. Health Sci Edu.

[8] Cruess SR, Cruess RL, Steinert Y (2008). Role modelling: making the most of a powerful teaching strategy. BMJ.

[9] Passi V, Johnson S, Piele E, Wright S, Hafferty F, Johnson N (2013). Doctor role modelling in medical education: BEME guide no. 27. Med Teach.

[10] Savin-Baden M, Howell Major C (2013). Qualitative research: the essential guide to theory and practice.

[11] O’Reilly M, Parker N (2013). Unsatisfactory saturation a critical exploration of the notion of saturated sample sizes in qualitative research. Qual Res.

[12] Corbin J, Strauss A (2015). Basics of qualitative research.

[13] Braun V, Clarke V (2006). Using thematic analysis. Qual Res Psychol.

[14] Lave J, Wenger E (1991). Situated learning*.* Legitimate peripheral participation.

[15] Gioia DA, Manz CC (1985). Linking cognition and behaviour: a script processing interpretation of vicarious learning. Acad Manag Rev.

[16] Ausubel DP (1960). The use of advance organizers in the learning and retention of meaningful verbal material. J Edu Psy.

[17] Bleakley A (2002). Pre-registration house officers & ward-based learning: ‘new apprenticeship model’ model Med Ed36.

[18] Nicol D (2010). From monologue to dialogue: improving written feedback processes in mass higher education. Assess Eval Higher Educ.

[19] Bucher R, Stelling JG (1977). Becoming professional.

[20] Biggs JB, Tang C (2011). Teaching for Quality Learning at University (4^th^ Ed.).

