# Supplementary material for: A skill to be worked at: using social learning theory to explore the process of learning from role models in clinical settings
Source: BMC Med Educ. 2018 Jul 3;18:156. doi: 10.1186/s12909-018-1251-x (PMC6029173; doi:10.1186/s12909-018-1251-x)
Supplement: Supplementary file 1 — Interview schedule – interview questions for both student and clinical teacher participants. (DOCX 13 kb) [file 12909_2018_1251_MOESM1_ESM.docx]

Additional file 1 - Interview schedule for students and clinical teachers

**Questions for students**

Give an example of a role model – what made you notice them?

What do you think you are learning/have learnt from role models?

How do you learn from role models in your clinical placements?

Do you do anything to help yourself learn from role models?

How have you put into practice what you have learnt from them?

What do role models to that help you to learn from them?

What makes it difficult to learn from role models?

**Questions for clinical teachers**

What do students learn from role models?

How do you think students learn from role models in this clinical placement?

What do you do to help students learn from you as a role model?

What could you do?

What makes it difficult for students to learn from role models?

How have you previously learnt from role models?

How do you currently learn from role models?
